# Severe Intraocular Inflammation Mimicking Masquerade Syndrome in a Patient Treated with Dupilumab: A Case Report

**DOI:** 10.1159/cop/acaag004

**Published:** 2026-08-18

**Authors:** Lorenzo Hu, Andrea Scupola, Carmela Grazia Caputo, Monica Maria Pagliara, Francesco Mottola, Giorgia Campaniello, Gustavo Savino, Claudia Fossataro, Maria Grazia Sammarco

**Affiliations:** a Department of Ophthalmology, “Fondazione Policlinico Universitario A. Gemelli, IRCCS”, Rome, Italy; b Department of Ophthalmology, Catholic University “Sacro Cuore”, Rome, Italy; c Ocular Oncology Unit, “Fondazione Policlinico Universitario A. Gemelli, IRCCS”, Rome, Italy

**Keywords:** Dupilumab, Intraocular inflammation, Uveitis, Exudative retinal detachment, Masquerade syndrome, Phthisis bulbi, Adverse drug reaction

## Abstract

**Introduction:**

The aim of the study was to report a case of severe vision-threatening intraocular inflammation with exudative retinal detachment presenting as a masquerade syndrome in a patient receiving dupilumab therapy for severe bronchial asthma.

**Case Presentation:**

A 51-year-old male with severe bronchial asthma developed sudden-onset no light perception (NLP) vision, iris rubeosis, pupillary seclusion, and total exudative retinal detachment in the right eye during his second cycle of dupilumab treatment. The clinical presentation raised strong suspicion for intraocular malignancy. Comprehensive evaluation including fluorescein and indocyanine green angiography, B-scan ultrasonography, magnetic resonance imaging, and positron emission tomography-computed tomography (PET-CT) was performed. PET-CT demonstrated intense metabolic activity in the right eye (SUV max 19.6) and a pulmonary nodule (SUV max 3.5). Lung biopsy revealed inflammatory changes only, and vitreous sampling excluded malignancy. Dupilumab was discontinued, and systemic and topical corticosteroids were administered. Despite treatment, the eye remained NLP. The course was further complicated by sinusitis and orbital cellulitis, requiring hospitalization. Over more than 12 months of follow-up, the right eye progressed to chronic hypotony and gradual phthisis bulbi. Application of the Naranjo Adverse Drug Reaction Probability Scale yielded a score of 6, classifying the association as probable.

**Conclusion:**

This case represents a severe dupilumab-associated ocular complication. Clinicians prescribing dupilumab should be vigilant for severe ocular adverse events beyond the commonly recognized conjunctivitis.

## Introduction

Dupilumab is a fully human monoclonal antibody targeting the interleukin-4 receptor alpha (IL-4Rα) subunit, blocking both IL-4 and IL-13 signaling pathways central to type 2 inflammation. It is approved for moderate-to-severe asthma, atopic dermatitis, chronic rhinosinusitis with nasal polyposis, eosinophilic esophagitis, and prurigo nodularis and has demonstrated significant efficacy across these indications [1, 2].

Despite a generally favorable safety profile, dupilumab has been associated with a spectrum of ocular adverse events. Conjunctivitis is most frequently reported, affecting 10–30% of patients, with additional manifestations including keratitis, blepharitis, dry eye, cicatrizing conjunctivitis, and punctal stenosis [3]. More rarely, cases of anterior and intermediate uveitis have been documented [4–6]. The pathophysiology is hypothesized to involve a shift from Th2- to Th1/Th17-predominant inflammation, potentially disrupting ocular immune homeostasis [7, 8].

While anterior segment complications are increasingly recognized, severe posterior segment inflammation with vision-threatening consequences remains exceptionally rare. After conducting a literature review in February 2026 utilizing PubMed, Google Scholar, and Scopus using the keywords dupilumab, intraocular inflammation, uveitis, masquerade syndrome, retinal detachment, and adverse drug reaction, we did not find any prior reports of dupilumab-associated intraocular inflammation presenting with complete vision loss, massive exudative retinal detachment, and imaging features mimicking intraocular malignancy, constituting a masquerade syndrome.

## Case Presentation

Written informed consent was obtained from the patient for publication of this case report and accompanying images. The CARE Checklist has been completed by the authors for this case report, attached as online supplementary material (for all online suppl. material, see https://doi.org/10.1159/cop/acaag004).

### Patient Information

A 51-year-old Caucasian male was referred to the Ocular Oncology Unit at Fondazione Policlinico Universitario A. Gemelli, Rome, Italy, for evaluation of a presumed masquerade syndrome. His medical history was significant for severe bronchial asthma requiring biologic therapy. Approximately 1 year before presentation, he had completed an initial cycle of 12 dupilumab injections (300 mg subcutaneously every 15 days) with satisfactory control of respiratory symptoms. His pulmonologist subsequently recommended a second cycle of six injections, during which the patient developed acute ocular symptoms. Dupilumab was discontinued on November 1, 2023.

### Clinical Presentation

The patient reported rapid deterioration of vision in the right eye over 3 weeks, accompanied by ocular pain, periorbital swelling, and redness. He denied any history of ocular trauma, prior uveitis, systemic autoimmune disease, immunosuppression, or known malignancy. There was no history of recent travel or tuberculosis exposure.

### Clinical Findings

At the initial examination on November 28, 2023, best-corrected visual acuity was no light perception (NLP) in the right eye and 20/20 in the left eye. Intraocular pressure was digitally low in the right eye and 15 mm Hg in the left eye. Slit-lamp examination of the right eye revealed marked iris rubeosis, complete pupillary seclusion, and a dense cataract precluding adequate fundoscopic visualization (Fig. 1). Fundus examination through the media opacities demonstrated total exudative retinal detachment and an amelanotic lesion at the posterior pole. Optical coherence tomography could not be performed due to media opacities. The left eye examination was unremarkable.

**Fig. 1.**
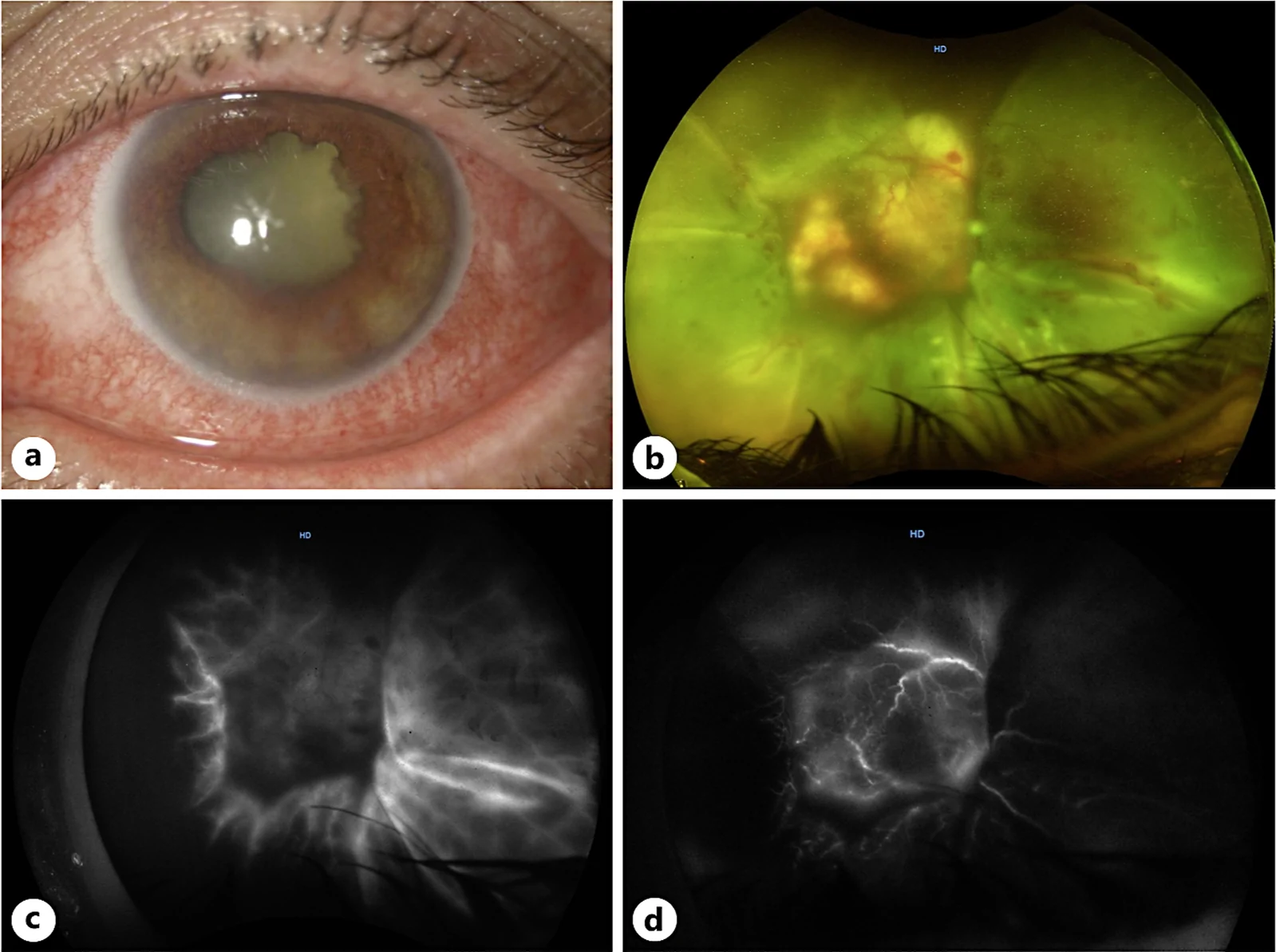
Anterior segment photograph and fundus angiography of the right eye. **a** Slit-lamp photograph demonstrating iris rubeosis and pupillary seclusion. **b** Fundus photograph showing total exudative retinal detachment with an amelanotic lesion at the posterior pole. **c**, **d** Fluorescein angiography and indocyanine green angiography demonstrating widespread vasculitic phenomena and disruption of the inner blood-retinal barrier.

### Diagnostic Assessment

Fluorescein angiography and indocyanine green angiography of the right eye, despite suboptimal image quality, demonstrated anterior displacement of the neuroretinal complex secondary to massive exudative detachment, widespread vasculitic phenomena, and extensive disruption of the inner blood-retinal barrier (Fig. 1). B-scan ultrasonography revealed dense vitreous opacities, total retinal detachment with increased subretinal corpuscular density, thickening of the sub-Tenon’s space inferiorly, and a solid heterogeneous thickening at the posterior pole raising concern for an intraocular mass (Fig. 2).

**Fig. 2.**
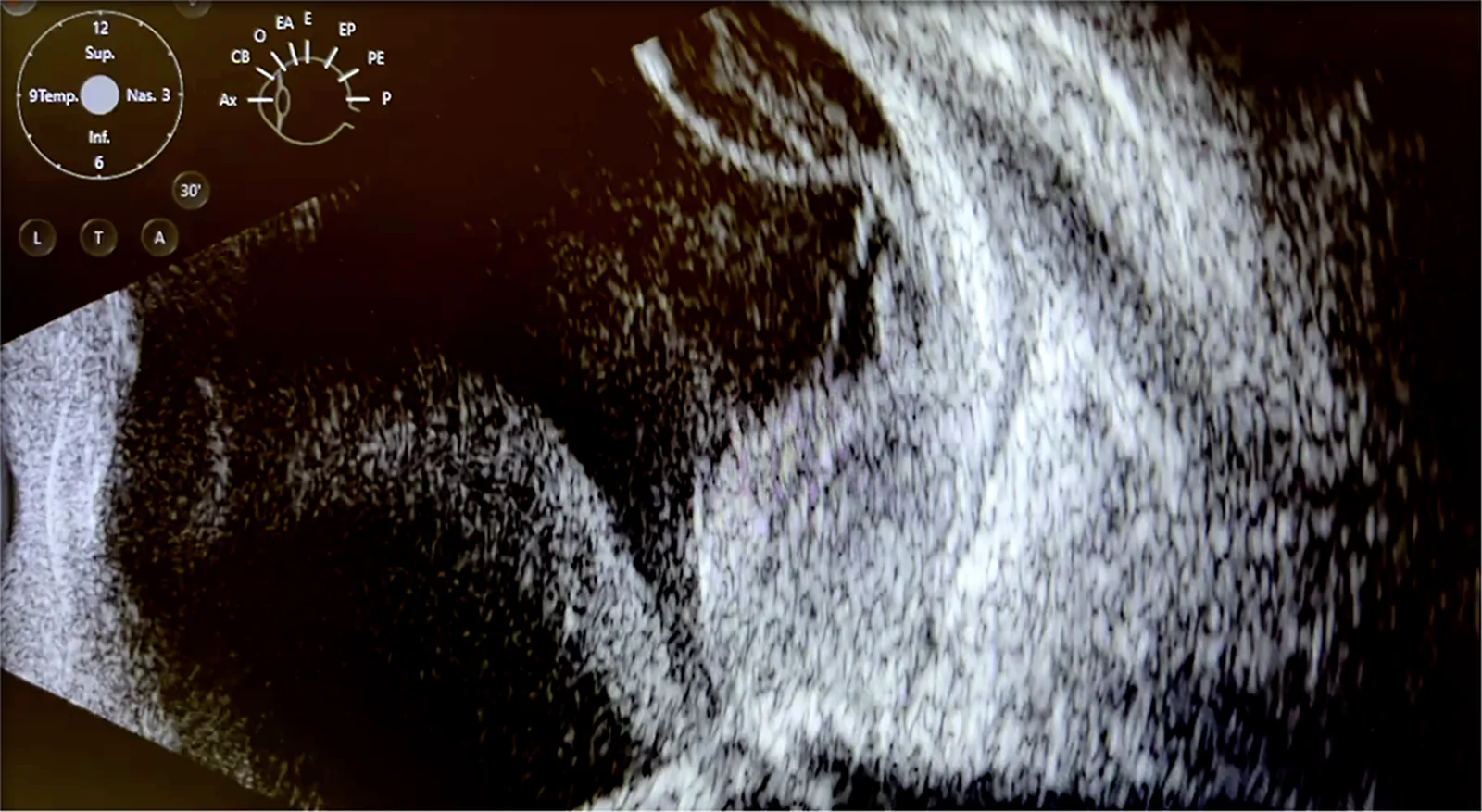
B-scan ultrasonography of the right eye demonstrating total retinal detachment with increased subretinal corpuscular density, vitreous opacities, and solid heterogeneous thickening at the posterior pole.

Magnetic resonance imaging of the orbits demonstrated a heterogeneous infiltrative lesion in the peripapillary region with massive retinal detachment and diffuse ocular wall enhancement, highly suspicious for intraocular malignancy (Fig. 3). Positron emission tomography-computed tomography (PET-CT) revealed intense fluorodeoxyglucose uptake in the right eye (SUV max 19.6), concentrated posteroinferiorly and laterally, and a 7-mm pulmonary nodule in the right upper lobe with moderate metabolic activity (SUV max 3.5). No systemic malignancy or lymphadenopathy was identified.

**Fig. 3.**
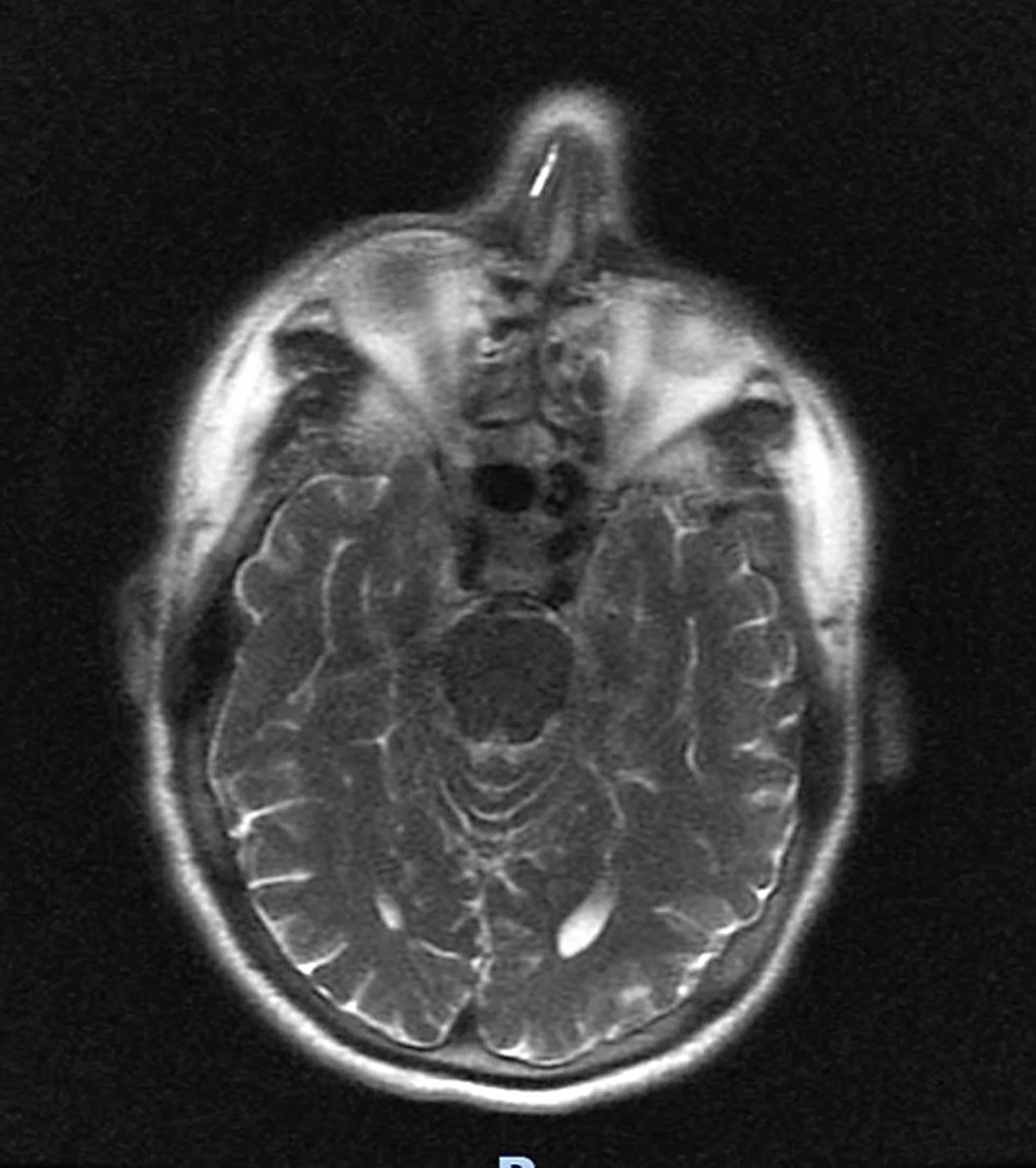
Magnetic resonance imaging of the orbits showing a heterogeneous infiltrative lesion in the peripapillary region of the right eye with massive retinal detachment and diffuse ocular wall enhancement.

Laboratory investigations including complete blood count, erythrocyte sedimentation rate, C-reactive protein, liver and renal function tests, QuantiFERON-TB Gold, serum angiotensin-converting enzyme, antinuclear antibodies, antineutrophil cytoplasmic antibodies, and serologies for syphilis, toxoplasmosis, Bartonella, Lyme disease, and HIV were all normal or negative, except for mild elevation of erythrocyte sedimentation rate and C-reactive protein.

CT-guided biopsy of the pulmonary nodule revealed chronic inflammatory infiltrate without evidence of malignancy or granulomatous disease. Diagnostic pars plana vitreous sampling for cytological, microbiological, and molecular analysis yielded insufficient cellular material for definitive diagnosis; no malignant cells, infectious organisms, or monoclonal lymphocyte populations were identified.

Approximately 2 to 4 weeks following the diagnostic pars plana vitreous biopsy, the patient developed ipsilateral acute rhinosinusitis complicated by orbital cellulitis, in the context of ongoing systemic corticosteroid therapy. The eye was already NLP at the time of this complication. The patient was hospitalized and managed with intravenous broad-spectrum antibiotics, with resolution of the orbital infection. Sinus and orbital cultures were not obtained or yielded no growth.

### Therapeutic Intervention and Follow-Up

Dupilumab had been discontinued on November 1, 2023, prior to referral, in consultation with the treating pulmonologist. Systemic corticosteroid therapy was initiated along with topical prednisolone acetate 1% and cycloplegic agents for the right eye. Despite treatment, the intraocular inflammation proved refractory, with vision remaining NLP. The clinical course was further complicated by the episode of orbital cellulitis secondary to ipsilateral sinusitis, which required hospitalization and intravenous antibiotic therapy. Although the orbital infection resolved, it likely contributed to the progressive structural deterioration of the already-compromised globe. Over more than 12 months of follow-up, the right eye progressed to chronic hypotony and gradual phthisis bulbi.

## Discussion

This case describes a severe ocular complication associated with dupilumab therapy. The presentation was characterized by NLP vision, iris rubeosis, pupillary seclusion, massive exudative retinal detachment, and intense fluorodeoxyglucose uptake on PET-CT (SUV max 19.6), strongly mimicking intraocular malignancy. Despite dupilumab discontinuation and corticosteroid therapy, the eye remained NLP. The clinical course was further complicated by sinusitis and orbital cellulitis, likely facilitated by concurrent systemic immunosuppression, which may have contributed to the progression toward phthisis bulbi. The clinical and imaging features necessitated systematic exclusion of masquerade syndromes. Primary vitreoretinal lymphoma was considered given the intense PET-CT uptake, vitritis, and unilateral presentation but was excluded by negative vitreous cytology and absence of CNS lesions. Uveal melanoma was excluded based on atypical imaging characteristics (diffuse wall thickening rather than focal mass) and negative biopsy. Metastatic carcinoma was considered given the concurrent pulmonary nodule; however, lung biopsy demonstrated inflammatory tissue only. Infectious etiologies (tuberculosis, syphilis, toxoplasmosis) were excluded by comprehensive serological testing. Vogt-Koyanagi-Harada disease was deemed unlikely given the strictly unilateral involvement and absence of extraocular manifestations. Sarcoidosis was considered, but the lung biopsy was non-granulomatous and serum angiotensin-converting enzyme was normal [9].

We applied the Naranjo Adverse Drug Reaction Probability Scale to systematically evaluate the likelihood that dupilumab caused the intraocular inflammation [10]. Points were assigned as follows: previous reports of dupilumab-associated uveitis exist (+1); the adverse event appeared after drug administration (+2); there was no improvement after discontinuation (0); no rechallenge was performed (0); alternative causes were comprehensively excluded (+2); the event was confirmed by objective evidence (+1). The total Naranjo score of 6 classifies the association as a probable adverse drug reaction. Notably, the score would have been higher had the inflammation resolved upon drug withdrawal; the refractory course may reflect the severity of the inflammatory insult rather than weakening the causal association. The development of ipsilateral sinusitis with orbital cellulitis following the diagnostic vitreous biopsy introduces an additional factor in the clinical trajectory. Importantly, the eye had already reached NLP with established exudative retinal detachment and neovascular features before the infectious complication occurred, confirming that the primary vision-threatening damage was attributable to the intraocular inflammatory process rather than the orbital infection. The cellulitis likely resulted from a convergence of risk factors: surgical disruption of the orbital barrier by vitreous biopsy, systemic corticosteroid-induced immunosuppression, and a severely inflamed ipsilateral globe. While the orbital cellulitis may have accelerated the structural deterioration toward phthisis bulbi, it does not alter the Naranjo causality assessment for the dupilumab-inflammation association as the irreversible visual outcome was established prior to the infectious episode.

Dupilumab-associated intraocular inflammation has been described in a limited number of reports. Padidam et al. [4] reported four cases, including posterior scleritis, anterior/intermediate uveitis, and cystoid macular edema, all improving after dupilumab cessation. Achten et al. [5] described 5 patients with non-infectious uveitis showing a pro-inflammatory Th1/Th17 molecular profile. Zhang et al. [6] reported bilateral panuveitis with hypereosinophilia, and Ayasse et al. [11] documented uveitis recurrence upon rechallenge, providing strong evidence of causality. Our case differs substantially from all published reports in severity (NLP vs. mild-to-moderate visual impairment), anatomical extent (massive exudative retinal detachment vs. limited findings), masquerade presentation requiring extensive oncological investigation, neovascular complications (iris rubeosis), and irreversible outcome (phthisis bulbi vs. improvement in all prior cases).

The concept of biologic-induced severe intraocular inflammation has precedent in the immune checkpoint inhibitor (ICI) literature. Checkpoint inhibitors targeting PD-1/PD-L1 and CTLA-4 are associated with uveitis in 2–4% of treated patients, occasionally presenting as VKH-like disease with exudative retinal detachments [12–15]. Although the mechanisms differ, ICIs disinhibit global T-cell activation, whereas dupilumab selectively blocks Th2 signaling; both drug classes can disrupt ocular immune homeostasis, producing severe posterior segment inflammation. This parallel underscores that biologic agents modulating immune pathways carry inherent risk for vision-threatening ocular inflammation.

The biological plausibility of dupilumab-induced intraocular inflammation rests on several mechanisms. First, by blocking IL-4/IL-13 signaling, dupilumab may disinhibit Th1 pathway activation, upregulating IFN-γ and TNF-α, cytokines strongly implicated in non-infectious uveitis. IL-4 and IL-13 have been shown to exert protective anti-inflammatory effects in experimental uveitis models, and their blockade may remove this protective brake [7, 15, 16]. Second, the eye maintains immune privilege partly through IL-4/IL-13-mediated suppression; disruption of this balance may precipitate breakdown of immune tolerance. Third, paradoxical hypereosinophilia reported with dupilumab may contribute to tissue damage through eosinophil infiltration [6]. Finally, individual genetic susceptibility, including polymorphisms in immune regulatory genes, may render certain patients vulnerable to severe ocular inflammation.

This case carries important implications for clinical practice. Patients initiating dupilumab should receive comprehensive counseling about ocular adverse events, including rare but severe vision-threatening complications beyond the commonly discussed conjunctivitis. Any visual symptoms, including blurring, pain, redness, and floaters, should prompt urgent ophthalmological referral rather than being attributed to expected ocular surface disease [17]. In severe or atypical presentations, a systematic approach to exclude neoplastic and infectious masquerade syndromes is essential, including multimodal imaging and vitreous biopsy when indicated. Multidisciplinary collaboration between ophthalmology and the prescribing specialist is critical for managing these complex cases.

Strengths of this report include comprehensive diagnostic evaluation systematically excluding alternative diagnoses, well-documented temporal association, multimodal imaging characterization, standardized causality assessment, and extended follow-up exceeding 12 months. Limitations include the inherent constraints of a single case report, inconclusive vitreous biopsy precluding histopathological characterization, absence of aqueous or vitreous cytokine profiling, and the development of post-procedural orbital cellulitis that may have contributed to the progression toward phthisis, confounding the assessment of the natural history of dupilumab-associated inflammation alone.

## Conclusions

This case report describes the most severe presentation of dupilumab-associated intraocular inflammation reported to date, characterized by complete vision loss, massive exudative retinal detachment, features mimicking intraocular malignancy on PET-CT. Unlike previously published cases of dupilumab-associated uveitis, which showed improvement after drug discontinuation, this case demonstrates that rare but devastating, irreversible outcomes can occur. Clinicians and patients should be aware that dupilumab-associated ocular complications extend beyond conjunctivitis to include severe posterior segment inflammation with potential for permanent vision loss. Pharmacovigilance reporting and further research into risk factors and pathophysiological mechanisms are essential.

## Acknowledgments

The authors thank the Italian Ministry of Health for support through Ricerca Corrente 2026.

## Statement of Ethics

This study was performed in accordance with the Declaration of Helsinki. Ethics approval for this human study was waived by the Catholic University of the Sacred Heart Ethical Committee in Rome, Italy. All adult participants provided written informed consent to participate in this study. Written informed consent was obtained from the individual for publication of the details of their medical case and any accompanying images. The authors have completed the CARE reporting checklist.

## Conflict of Interest Statement

The authors have no conflicts of interest to declare.

## Author Contributions

L.H. was responsible for manuscript drafting, literature review, and data analysis. M.M.P. and M.G.S. contributed to patient management, data collection, and manuscript revision. M.M.P. and G.S. provided clinical assessment, imaging interpretation, and manuscript revision. A.S., C.G.C., F.M., G.C., and C.F. contributed to data collection and clinical oversight. All authors have read and approved the final manuscript.

## Data Availability

The data that support the findings of this study are not publicly available due to patient privacy and confidentiality reasons but are available from the corresponding author upon reasonable request.
